# Deriving utility scores for co-morbid conditions: a test of the multiplicative model for combining individual condition scores

**DOI:** 10.1186/1478-7954-4-13

**Published:** 2006-10-31

**Authors:** William Flanagan, Cameron N McIntosh, Christel Le Petit, Jean-Marie Berthelot

**Affiliations:** 1 Health Analysis and Measurement Group, Statistics Canada, R.H. Coats Building, Ottawa, Ontario, K1A 0T6, Canada

## Abstract

**Background:**

The co-morbidity of health conditions is becoming a significant health issue, particularly as populations age, and presents important methodological challenges for population health research. For example, the calculation of summary measures of population health (SMPH) can be compromised if co-morbidity is not taken into account. One popular co-morbidity adjustment used in SMPH computations relies on a straightforward multiplicative combination of the severity weights for the individual conditions involved. While the convenience and simplicity of the multiplicative model are attractive, its appropriateness has yet to be formally tested. The primary objective of the current study was therefore to examine the empirical evidence in support of this approach.

**Methods:**

The present study drew on information on the prevalence of chronic conditions and a utility-based measure of health-related quality of life (HRQoL), namely the Health Utilities Index Mark 3 (HUI3), available from Cycle 1.1 of the Canadian Community Health Survey (CCHS; 2000–01). Average HUI3 scores were computed for both single and co-morbid conditions, and were also *purified *by statistically removing the loss of functional health due to health problems other than the chronic conditions reported. The co-morbidity rule was specified as a multiplicative combination of the purified average observed HUI3 utility scores for the individual conditions involved, with the addition of a synergy coefficient *s *for capturing any interaction between the conditions not explained by the product of their utilities. The fit of the model to the purified average observed utilities for the co-morbid conditions was optimized using ordinary least squares regression to estimate *s*. Replicability of the results was assessed by applying the method to triple co-morbidities from the CCHS cycle 1.1 database, as well as to double and triple co-morbidities from cycle 2.1 of the CCHS (2003–04).

**Results:**

Model fit was optimized at *s *= .99 (i.e., essentially a straightforward multiplicative model). These results were closely replicated with triple co-morbidities reported on CCHS 2000–01, as well as with double and triple co-morbidities reported on CCHS 2003–04.

**Conclusion:**

The findings support the simple multiplicative model for computing utilities for co-morbid conditions from the utilities for the individual conditions involved. Future work using a wider variety of conditions and data sources could serve to further evaluate and refine the approach.

## Background

Over the past century, advances in both public and population health have dramatically increased life expectancy in many parts of the developed world. However, these improvements in life expectancy may be accompanied by higher morbidity due to the increased presence of chronic conditions. Indeed, the phenomenon of co-morbidity – the clustering of different health conditions within individuals – is quite common as populations age [1,2]. A substantial amount of empirical research has shown that the number of co-morbid conditions experienced by patients is positively associated with mortality risk, utilization of health care services, and decrements in health-related quality of life (HRQoL) [3,4]. Given this considerable economic and HRQoL impact of co-morbidity, and also that the proportion of those aged 65 and over is expected to increase substantially in many developed countries over the next two decades [5], it is not surprising that co-morbidity has been identified as a key research priority by a number of researchers [3,6]. For those examining this issue, quantitative methods for handling co-morbidity are essential, in order to avoid bias when generating various indices of the impact of chronic and other conditions [7].

Adjusting for co-morbidity is particularly important in the computation of summary measures of population health (SMPH) that combine information on mortality and morbidity [8]. Over the past 15 years, there has been a steady increase in methodological sophistication for dealing with co-morbidity in SMPH calculations. In the original Global Burden of Disease (GBD) study conducted by the World Health Organization (WHO) and its collaborators in 1990, co-morbidity resulted in overestimation of total disability-adjusted life years (DALYs), since the severity-weighted prevalence of various specific conditions was simply summed as part of the overall burden calculation [9]. Researchers in the Netherlands also adopted this methodology [10]. However, Murray and Lopez have since acknowledged that the additive approach to co-morbidity used in the GBD 1990 was overly simplistic and implausible [11].

Recognizing such issues, an alternative approach was undertaken for the DALY calculations in the Australian [12] and Victorian [13] Burden of Disease studies. Specifically, in order to adjust for the co-morbidity of prevalent mild conditions in older age groups, the severity weights for the individual conditions were combined *a priori *using a multiplicative model. Given its simplicity and ease of interpretation, the multiplicative "rule" for combining severity weights for individual conditions continues to be used when adjusting SMPH for co-morbidity [14-16]; however, its appropriateness has not yet been verified empirically. Mathers et al. [17] concluded that until research directly addresses how severity weights should be combined, the multiplicative approach appears reasonable. However, as Schneeweis et al. [18] have noted, there currently exists no "gold standard" method or measure for dealing with co-morbidity, with most being selected for "convenience rather than performance." Therefore, it is important to subject the co-morbidity methods that have been proposed to more rigorous empirical testing, in order to better determine their effectiveness and identify areas for potential refinement. Such an approach will assist in improving ongoing efforts to develop SMPH attributable to individual conditions [19]; in particular, it would contribute to a more precise rank ordering of conditions in terms of their population health impact, thereby better informing health policy decisions concerning the allocation of scarce societal resources to prevention and treatment programs.

With these considerations in mind, the present study specifically tested the extent to which a multiplicative model for combining the health state utilities for individual conditions could reproduce the utility for the co-morbidity of the same conditions (see Appendix A, Note 1). To meet this objective, this study used self-reported information on both HRQoL and the prevalence of chronic conditions in Canada from a nationally representative population health survey.

## Methods

### Functional form of the co-morbidity rule

In its simplest form, a multiplicative model for combining the utilities of two or more distinct conditions *u*_1 _and *u*_2 _to generate a theoretical utility u^
 MathType@MTEF@5@5@+=feaafiart1ev1aaatCvAUfKttLearuWrP9MDH5MBPbIqV92AaeXatLxBI9gBaebbnrfifHhDYfgasaacH8akY=wiFfYdH8Gipec8Eeeu0xXdbba9frFj0=OqFfea0dXdd9vqai=hGuQ8kuc9pgc9s8qqaq=dirpe0xb9q8qiLsFr0=vr0=vr0dc8meaabaqaciaacaGaaeqabaqabeGadaaakeaacuWG1bqDgaqcaaaa@2E2F@_1,2 _for their co-morbidity is written as follows:

u^
 MathType@MTEF@5@5@+=feaafiart1ev1aaatCvAUfKttLearuWrP9MDH5MBPbIqV92AaeXatLxBI9gBaebbnrfifHhDYfgasaacH8akY=wiFfYdH8Gipec8Eeeu0xXdbba9frFj0=OqFfea0dXdd9vqai=hGuQ8kuc9pgc9s8qqaq=dirpe0xb9q8qiLsFr0=vr0=vr0dc8meaabaqaciaacaGaaeqabaqabeGadaaakeaacuWG1bqDgaqcaaaa@2E2F@_1,2 _= *u*_1 _* *u*_2 _    [1]

Such a model has three desirable properties. First, for health utilities between 0 (death) and 1 (full health), their product is also bounded by 0 and 1. A second and intuitively appealing property of the model is that the utility for the co-morbidity is proportional to the individual utilities associated with each of the individual conditions involved. In other words, each additional condition reduces functional health relative to its previous level. For example, those in full health who developed a condition having an associated utility of 0.90 would maintain 90% of full functional health, that is, a utility of 0.90. If they developed a second condition with utility 0.80, their utility for their functional health state would be reduced to 0.72 (i.e., .80 * .90). Third, a multiplicative model can be applied to the utilities for any number of conditions, and is commutative.

Since the purpose of the present study is to assess the appropriateness of the multiplicative model by examining how well it could reproduce observed utilities associated with co-morbid conditions, it is necessary to augment Equation [1] with a "synergy" coefficient *s*, as follows:

u^
 MathType@MTEF@5@5@+=feaafiart1ev1aaatCvAUfKttLearuWrP9MDH5MBPbIqV92AaeXatLxBI9gBaebbnrfifHhDYfgasaacH8akY=wiFfYdH8Gipec8Eeeu0xXdbba9frFj0=OqFfea0dXdd9vqai=hGuQ8kuc9pgc9s8qqaq=dirpe0xb9q8qiLsFr0=vr0=vr0dc8meaabaqaciaacaGaaeqabaqabeGadaaakeaacuWG1bqDgaqcaaaa@2E2F@_1,2 _= s * (*u*_1 _* *u*_2 _).     [2]

The inclusion of *s *in the formula allows the model to be adjusted to better fit the data; and therefore the value estimated for *s *can be used to judge the appropriateness of the multiplicative form. In particular, a synergy coefficient close to 1 would indicate that most of the utility associated with co-morbidity is explained by the straightforward multiplication of the utilities for the separate conditions, showing that the simple multiplicative form is appropriate. On the other hand, a synergy coefficient far from 1 would indicate that most of the utility linked to co-morbidity is explained by this coefficient. In this case, one would conclude that there is additional interaction among conditions not adequately accounted for by the simple multiplicative model.

### Data Source and Variables

To empirically evaluate the multiplicative model, it was first necessary to obtain information on both chronic condition prevalence and HRQoL, in order to link observed health state utilities to both single and co-morbid chronic conditions. This analysis used self-reported data on these variables from cycle 1.1 of the Canadian Community Health Survey (CCHS), conducted in 2000–01 [20]. The CCHS is an ongoing, cross-sectional survey that collects information on health status, health determinants, and health care utilization. It is representative of the Canadian household population aged 12 and over in all provinces and territories, and excludes populations on Indian Reserves, Canadian Forces Bases, and certain remote areas. The sample size was 131,535 respondents in this first cycle. In addition, in order to verify the findings obtained with the cycle 1.1 data, we conducted a validation study using cycle 2.1 of the CCHS, conducted in 2003–04. This dataset contained 45,101 respondents who were asked questions related to the analysis variables of interest here.

Regarding the distribution of chronic conditions in the population, respondents to the CCHS cycle 1.1 were asked to indicate if they had any of 27 specific chronic conditions (Table 1), defined as "conditions that have lasted or are expected to last six months or more and have been diagnosed by a health professional." For the measure of HRQoL, the Health Utilities Index Mark 3 (HUI3) was selected [21,22]. The HUI3 is a derived variable on the CCHS database and is based on a respondent's standing on eight underlying health status attributes: Vision, Hearing, Speech, Ambulation, Dexterity, Emotion, Cognition, and Pain. Each attribute has five to six levels ranging from normal to severely limited functioning. For example, the Ambulation attribute has levels which range from 1 ("Able to walk around the neighbourhood without difficulty, and without walking equipment") to 6 ("Unable to walk at all"). On the CCHS, respondents were asked a standardized set of questions on usual functional ability or capacity, which map to the levels on the eight attributes of the HUI3. Finally, the individual's scores across the eight attributes were combined using the HUI3 multi-attribute utility function in order to yield a global utility score representing the HRQoL of the respondent [23]. This score has a theoretical range of -0.36 to 1, where -0.36 and 1 represent the utilities of the worst and best possible HUI3 health states, respectively; and 0 represents death [22].

**Table 1:** Observed and purified HUI3 scores of 26 chronic conditions surveyed in cycle 1.1 of CCHS

**Condition reported alone on CCHS**	**Observed^Φ ^HUI3**	**Purified^† ^HUI3**
Non-Food Allergies	0.93 (0.00)	1.00 (0.00)
Thyroid Condition	0.93 (0.01)	1.00 (0.01)
Food Allergies	0.93 (0.01)	0.99 (0.01)
Cataracts	0.92 (0.01)	0.99 (0.01)
High Blood Pressure	0.92 (0.00)	0.98 (0.00)
Asthma	0.92 (0.01)	0.98 (0.01)
Suffers/Multiple Chemical Sensitivities	0.92 (0.02)	0.98 (0.02)
Diabetes	0.91 (0.01)	0.97 (0.01)
Heart Disease	0.90 (0.01)	0.97 (0.01)
Bowel Disorder-Crohn's Disease/Colitis	0.90 (0.01)	0.97 (0.01)
Migraine Headaches	0.90 (0.01)	0.97 (0.01)
Glaucoma	0.90 (0.02)	0.96 (0.03)
Stomach/Intestinal Ulcers	0.90 (0.01)	0.96 (0.01)
Chronic Bronchitis	0.89 (0.01)	0.96 (0.01)
Cancer	0.89 (0.02)	0.95 (0.02)
Epilepsy	0.88 (0.02)	0.94 (0.02)
Arthritis/Rheumatism	0.88 (0.01)	0.94 (0.01)
Back Problems*	0.88 (0.00)	0.94 (0.00)
Emphysema/COPD	0.87 (0.03)	0.93 (0.03)
Fibromyalgia	0.86 (0.02)	0.92 (0.02)
Chronic Fatigue Syndrome	0.81 (0.04)	0.87 (0.04)
Urinary Incontinence	0.76 (0.06)	0.81 (0.06)
Parkinson's Disease	0.75 (0.05)	0.80 (0.05)
Suffers From The Effects Of A Stroke	0.69 (0.08)	0.74 (0.08)
Multiple Sclerosis	0.69 (0.05)	0.74 (0.05)
Alzheimer's Disease/Other Dementia	0.45 (0.06)	0.48 (0.07)

Notes:

Conditions are shown in descending order of prevalence.

Standard error is shown in brackets following the estimate.

* Back problems excluding fibromyalgia & arthritis.

Φ *Observed *refers to the average, age-sex standardized HUI3 scores of the condition reported alone on CCHS before being *purified*.

† *Purified *refers to the average observed utility after division by the average utility of those reporting no conditions.

### Data preparation

All analyses were conducted using SAS version 9.1. Average HUI3 scores were computed for groups of persons reporting no, one or two chronic conditions, and were age- and sex-standardized to the Canadian population as represented by the survey sample weights (see Appendix A, Note 2) [24]. Further, to account for the complex sampling design of the CCHS, survey bootstrap weights were used to generate estimates of standard error (SE) around the mean estimates [25,26]. For the calculation of the average HUI3 associated with a pair of conditions, cases were excluded if they had missing values for the HUI3 score, or "unstated/unknown/refused" recorded for the chronic condition(s).

### Purification of HUI3 scores

Prior to estimating the model, another data preparation step involved *purifying *the average HUI3 scores in order to better reflect the HRQoL impact of the chronic conditions reported. A preliminary analysis revealed that the average HUI3 score of persons reporting no chronic conditions was less than full health: 0.94 (SE = 0.00). This may be due to conditions other than those specifically surveyed, such as influenza, or it may reflect a general state of health associated with ageing and not associated with any specific condition. Therefore, we postulated that for persons reporting one or more chronic conditions, part of their HUI3 score may also be attributable to these other factors. In other words, we have assumed that the derived HUI3 score on CCHS represented a co-morbidity of the condition(s) reported and these other factors. To avoid double-counting such effects when applying a co-morbidity rule, we *purified *all average HUI3 scores by removing the loss of functional health associated with these other factors. Specifically, purification was achieved by dividing the average HUI3 score for cases reporting one or more conditions by the average HUI3 score for those reporting no conditions. The resulting purified score is held to uniquely represent the utility associated with a given condition or combination of conditions, free of potential confounding due to additional unknown factors that impact functional health.

### Estimation of synergy coefficient s

The synergy coefficient that produced the best fit to the data was determined by applying the co-morbidity rule defined in Equation [2] across all co-morbid pairs of chronic conditions reported on the CCHS cycle 1.1. First, for each co-morbid pair, we computed a *theoretical *utility, namely the product of the *purified *average observed utilities for the individual conditions reported alone (see Equation [1]). Second, in order to estimate *s*, these theoretical utilities were fitted to the purified average observed utilities for the co-morbid pairs of chronic conditions, using ordinary least squares regression. The analysis was weighted according to the prevalence of the observed co-morbidities.

### Replicability

It is important to recognize that the optimal value obtained for the synergy coefficient *s *in the initial analysis might be sample-specific. Therefore, an additional objective was to determine the replicability of the results with respect to more than two co-morbid conditions, as well as in an independent data set. Thus, the co-morbidity rule was also applied to the set of respondents reporting three conditions on CCHS cycle 1.1, and to respondents reporting two and three chronic conditions on CCHS cycle 2.1.

## Results

### Descriptive statistics

The average age- and sex-standardized HUI3 score for persons reporting no chronic conditions was 0.94 (SE = 0.00). Thus a loss of utility of 0.06 was assumed to be associated with factors other than those reported by the respondents, and was used to purify the average HUI3 scores of persons reporting one or more conditions. Persons reporting one chronic condition had average age- and sex-standardized HUI3 scores ranging from 0.45 (SE = 0.06) to 0.93 (SE = 0.00); their purified HUI3 ranged from 0.48 (SE = 0.07) to 1.00 (SE = 0.00) (see Table 1). Persons that reported two chronic conditions in CCHS 2000–01, had average age- and sex-standardized HUI3 scores ranging from -0.01 (SE = 0.00) to 1.00 (SE = 0.00). However, the majority of these co-morbid conditions were relatively mild; specifically, of the 278 pairs of two conditions reported together on CCHS, 184 had average HUI3 scores above 0.80, and this accounted for 90% of the prevalence of all persons reporting two chronic conditions together.

### Estimation of synergy coefficient s

The synergy coefficient was estimated at *s *= 0.99, *t*(1) = 474.16 (*p *< .0001); the purified average observed utilities for the co-morbid pairs were very closely reproduced by the model, *R*^2 ^= 0.99, *F*(1, 277) = 224828, (*p *< .0001). Thus, the final form of the general co-morbidity rule for combining the utilities of two separate conditions to estimate the utility of their co-morbidity was the simple multiplicative model in Equation [1]. Table 2 shows the average observed HUI3, the purified HUI3, and the theoretical utility estimated from the multiplicative rule for the 20 most prevalent pairs of co-morbid conditions on the CCHS.

**Table 2:** Observed, purified and theoretical estimates of health utility for the 20 most prevalent co-morbid conditions (descending) in cycle 1.1 of CCHS

**Two conditions reported together on CCHS**		**Observed^Φ ^HUI3**	**Purified^† ^HUI3**	**Theoretical^‡ ^utility**
Non-Food Allergies	Asthma	0.93 (0.01)	1.00 (0.01)	0.98 (0.01)
Non-Food Allergies	Back Problems*	0.86 (0.01)	0.92 (0.01)	0.94 (0.01)
Food Allergies	Non-Food Allergies	0.92 (0.01)	0.99 (0.01)	0.99 (0.01)
Arthritis/Rheumatism	Back Problems*	0.78 (0.01)	0.83 (0.01)	0.88 (0.01)
Non-Food Allergies	Migraine Headaches	0.91 (0.01)	0.98 (0.01)	0.97 (0.01)
Arthritis/Rheumatism	High Blood Pressure	0.85 (0.01)	0.91 (0.01)	0.93 (0.01)
Non-Food Allergies	Arthritis/Rheumatism	0.83 (0.02)	0.89 (0.02)	0.94 (0.01)
Back Problems*	Migraine Headaches	0.82 (0.01)	0.88 (0.01)	0.91 (0.01)
Non-Food Allergies	High Blood Pressure	0.92 (0.01)	0.98 (0.01)	0.98 (0.01)
Back Problems*	High Blood Pressure	0.86 (0.02)	0.92 (0.02)	0.92 (0.01)
High Blood Pressure	Diabetes	0.89 (0.02)	0.96 (0.02)	0.96 (0.01)
Non-Food Allergies	Thyroid Condition	0.92 (0.01)	0.99 (0.01)	1.00 (0.01)
High Blood Pressure	Heart Disease	0.91 (0.02)	0.97 (0.02)	0.95 (0.01)
Arthritis/Rheumatism	Migraine Headaches	0.84 (0.02)	0.90 (0.02)	0.91 (0.01)
Food Allergies	Back Problems*	0.88 (0.01)	0.94 (0.01)	0.93 (0.01)
Asthma	Back Problems*	0.87 (0.02)	0.93 (0.02)	0.92 (0.01)
High Blood Pressure	Migraine Headaches	0.84 (0.02)	0.90 (0.02)	0.95 (0.01)
Food Allergies	Asthma	0.92 (0.01)	0.98 (0.02)	0.97 (0.01)
Arthritis/Rheumatism	Thyroid Condition	0.90 (0.02)	0.96 (0.02)	0.94 (0.01)
Food Allergies	Migraine Headaches	0.91 (0.01)	0.98 (0.02)	0.96 (0.01)

Notes:

Standard error is shown in brackets following the estimate.

Conditions are shown in descending order of prevalence of the observed co-morbidity.

* Back problems excluding fibromyalgia & arthritis.

Φ *Observed *refers to the average, age-sex standardized HUI3 of the pair of conditions reported together on CCHS before being *purified*.

† *Purified *refers to the average observed utility after division by the average utility of those reporting no conditions.

‡ *Theoretical *refers to the utility estimated from the multiplicative rule (no synergy), using the purified HUI3 of the two conditions reported alone (Table 1).

### Replicability

The co-morbidity rule was also tested for persons reporting three conditions (*n *= 924) on CCHS cycle 1.1: the synergy coefficient that best fit this dataset was *s *= 0.99. When the rule was tested on the next wave of CCHS (cycle 2.1 conducted in 2003–04) for two conditions (*n *= 299) and for three conditions (*n *= 734), the synergy coefficient yielding the best fit to the data was also *s *= 0.99 in both cases.

## Discussion and conclusions

The purpose of this study was to test a conventionally used multiplicative model for computing utilities for co-morbid conditions from the utilities for the individual conditions involved. Using information on the distribution of chronic conditions from a nationally representative general population health survey (CCHS 2000–01, cycle 1.1), as well as a reliable and well-validated utility-based measure of HRQoL (HUI3), it was found that a straightforward multiplicative model best suited the calculation of average utilities for 278 pairs of co-morbid conditions. Specifically, a synergy coefficient *s*, applied to the straightforward multiplicative model in order to allow best fit to the data, optimized model fit at a value of 0.99. This result shows that the utility linked to co-morbidity is adequately explained via simple multiplication of the utilities for the individual conditions; in other words, there appears to be no synergistic effect of having two or more conditions. Further, these results were closely replicated with respect to three co-morbid conditions and in an independent sample of data from the CCHS 2003–04 (cycle 2.1), suggesting that the initial synergy coefficient estimate was reliable and not just a sample-specific result or an artifact of the survey methodology. This study is the first to provide empirical confirmation of the multiplicative model commonly used to adjust for co-morbidity in the computation of SMPH [12-14,17].

The current support for this convenient and generalizable co-morbidity rule is particularly encouraging, given that other proposed methods for obtaining weights for co-morbid conditions might be practically difficult to implement. With regard to the original GBD 1990 project, Murray [27] had suggested that another potentially viable strategy for dealing with co-morbidity in future work might be to generate unique weights for particular condition combinations, using further health state valuation exercises (i.e., elicit the weights for co-morbidities directly from panels of raters). Andrews and his colleagues [28] also support this approach and note that empirical research on co-morbidities could add important information to the case descriptions used for the valuation exercises. This particular strategy has not yet been implemented, however, likely due to the considerable resources required to perform valuations of all possible co-morbidities. Further, any co-morbidities not considered in the initial valuation exercises, yet deemed relevant in later studies, would necessitate assembling further valuation panels to obtain the weights. This study suggests that the more easily-applied multiplicative model for individual condition weights is reasonable for deriving co-morbid condition weights in burden of disease research.

A methodological strength of the present study is the purification strategy applied to the average observed HUI3 utilities for the single and co-morbid conditions. By removing the loss of functional health attributable to unknown factors, this approach helps provide a clearer picture of the actual HRQoL impact of the conditions studied. We therefore recommend using this approach in similar future work, in order to minimize bias when using utility measures to estimate the HRQoL consequences of different conditions.

Some limitations of the current study should also be noted. First, while the multiplicative co-morbidity rule performs well in the prediction of average utilities, it is not intended to be a predictor of individual outcomes (e.g., for a particular patient). However, within the area of SMPH, the use of averages is appropriate. Second, the majority of chronic conditions available for analysis from the CCHS were relatively mild, that is, most of them do not have a very large impact on functional health in terms of HUI3 scores. Thus it is not certain that the co-morbidity rule would hold with more severe combinations of conditions such as might be found within clinical and institutional populations, which are currently not part of the CCHS. To address this issue, future evaluations of the co-morbidity rule could utilize other information sources such as hospital administrative databases. A related point is that the prevalence estimates for chronic conditions were self-rather than clinician-reported on the CCHS. Thus it is possible that prevalence may have been underestimated and that the influence of unreported conditions on functional health may have affected estimates of the HUI3 utilities for the conditions studied [29,30]; however, the purification strategy used here may have offered some protection against this problem. Again, future work using clinical administrative databases might help rectify such issues.

Despite these limitations, this study makes an important contribution in that it provides the first empirical assessment of the multiplicative rule for producing co-morbid condition weights, and suggests that continued use of the model is reasonable for use in constructing SMPH. Future research incorporating some of the suggestions noted above would serve to further evaluate and refine the approach.

## List of Abbreviations

CCHS = Canadian Community Health Survey

HRQoL = Health-Related Quality of Life

HUI3 = Health Utilities Index Mark 3

GBD = Global Burden of Disease

WHO = World Health Organization

DALY = Disability-Adjusted Life Year

SMPH = Summary Measures of Population Health

## Competing interests

The author(s) declare that they have no competing interests.

## Authors' contributions

CLP and JMB provided the conceptual and methodological framework for the study; WF

performed the analyses and wrote the Methods and Results sections of the paper; CNM

wrote the Introduction and Discussion and Conclusion sections and prepared the final

version of the paper. All authors commented on various drafts of the paper.

## Appendix A

### Note 1: Utilities versus severity weights

A brief note of clarification is in order here. In the original GBD 1990 study and the Australian and Victorian versions, the impact on HRQoL of a given disease was represented by a "severity weight" ranging between 0 (full health) and 1 (death). In the current study, we quantify HRQoL in terms of health state utilities, which are essentially the complement of severity weights (i.e., 1 – severity weight). The reason for this approach is to maintain consistency with the HRQoL instrumentation used in the current study, namely the Health Utilities Index Mark 3 (HUI3), which represents HRQoL as utilities.

### Note 2: Method of age- and sex-standardization

All estimates of the average of the observed HUI3 utility scores for each subpopulation, defined by the type of chronic condition(s) reported, were age- and sex-standardized to the total Canadian population (age groups were 12–19, 20–29, 30–39, 40–49, 50–59, 60–69, 70–79, and 80+). This was accomplished in two main steps, as follows:

(1) the CCHS survey sample weights were re-scaled according to the ratio of the proportion of individuals in the total Canadian population in a specific age group and sex to the proportion of individuals in the subpopulation having the chronic condition(s) in the same age group and sex. This can be stated more formally:

*v*_*i*,*b *_= *w*_*i*,b _* *p*_*a*,*s *_/*y*_*a*,*s*,*b *_

where

• *v*_*i*,*b *_represents the age- and sex-standardized survey sample weight of the *i*^th ^record in the chronic condition subpopulation *b *(unitary or co-morbid);

• *w*_*i*,*b *_represents the survey sample weight of the *i*^th ^record in the chronic condition subpopulation *b*;

• *p*_*a*,*s *_represents the proportion of cases in the total Canadian population in age group *a *of sex *s*, and is defined as

pa,s=∑m=1Mwm/∑n=1Nwn,
 MathType@MTEF@5@5@+=feaafiart1ev1aaatCvAUfKttLearuWrP9MDH5MBPbIqV92AaeXatLxBI9gBaebbnrfifHhDYfgasaacH8akY=wiFfYdH8Gipec8Eeeu0xXdbba9frFj0=OqFfea0dXdd9vqai=hGuQ8kuc9pgc9s8qqaq=dirpe0xb9q8qiLsFr0=vr0=vr0dc8meaabaqaciaacaGaaeqabaqabeGadaaakeaacqWGWbaCdaWgaaWcbaGaemyyaeMaeiilaWIaem4Camhabeaakiabg2da9maaqahabaGaem4DaC3aaSbaaSqaaiabd2gaTbqabaGccqGGVaWldaaeWbqaaiabdEha3naaBaaaleaacqWGUbGBaeqaaaqaaiabd6gaUjabg2da9iabigdaXaqaaiabd6eaobqdcqGHris5aaWcbaGaemyBa0Maeyypa0JaeGymaedabaGaemyta0eaniabggHiLdGccqGGSaalaaa@4848@

where *w*_*m *_is the survey sample weight for the *m*^th ^of *M *records in age group *a *with sex *s*, and *w*_*n *_is the survey sample weight for the *n*^th ^of all *N *records in the entire CCHS survey data file;

• *y*_*a*,*s*,*b *_, represents the proportion of cases in age group *a *with sex *s *in the subpopulation *b *that have the chronic condition(s), and is defined exactly like *p*_*a*,*s *_except restricted to cases in the subpopulation *b*, that is:

ya,s,b=∑q=1Qwq/∑r=1Rwr
 MathType@MTEF@5@5@+=feaafiart1ev1aaatCvAUfKttLearuWrP9MDH5MBPbIqV92AaeXatLxBI9gBaebbnrfifHhDYfgasaacH8akY=wiFfYdH8Gipec8Eeeu0xXdbba9frFj0=OqFfea0dXdd9vqai=hGuQ8kuc9pgc9s8qqaq=dirpe0xb9q8qiLsFr0=vr0=vr0dc8meaabaqaciaacaGaaeqabaqabeGadaaakeaacqWG5bqEdaWgaaWcbaGaemyyaeMaeiilaWIaem4CamNaeiilaWIaemOyaigabeaakiabg2da9maaqahabaGaem4DaC3aaSbaaSqaaiabdghaXbqabaaabaGaemyCaeNaeyypa0JaeGymaedabaGaemyuaefaniabggHiLdGccqGGVaWldaaeWbqaaiabdEha3naaBaaaleaacqWGYbGCaeqaaaqaaiabdkhaYjabg2da9iabigdaXaqaaiabdkfasbqdcqGHris5aaaa@49C2@

where *w*_*q *_is the survey sample weight for the *q*^th ^of *Q *survey records in chronic condition subpopulation *b *in age group *a *with sex *s*, and *w*_*r *_is the survey sample weight for the *r*^th ^of all *R *survey records in the chronic condition subpopulation *b*.

(2) the individual HUI3 utility scores of persons reporting chronic condition(s) *b *were adjusted according to the age- and sex-standardized weights from step 1, to estimate average age- and sex-standardized HUI3 scores for each chronic condition, u¯
 MathType@MTEF@5@5@+=feaafiart1ev1aaatCvAUfKttLearuWrP9MDH5MBPbIqV92AaeXatLxBI9gBaebbnrfifHhDYfgasaacH8akY=wiFfYdH8Gipec8Eeeu0xXdbba9frFj0=OqFfea0dXdd9vqai=hGuQ8kuc9pgc9s8qqaq=dirpe0xb9q8qiLsFr0=vr0=vr0dc8meaabaqaciaacaGaaeqabaqabeGadaaakeaacuWG1bqDgaqeaaaa@2E37@_*b *_, as follows:

u¯b=∑i=1R(vi,b*ui,b)∑i=1Rvi,b,
 MathType@MTEF@5@5@+=feaafiart1ev1aaatCvAUfKttLearuWrP9MDH5MBPbIqV92AaeXatLxBI9gBaebbnrfifHhDYfgasaacH8akY=wiFfYdH8Gipec8Eeeu0xXdbba9frFj0=OqFfea0dXdd9vqai=hGuQ8kuc9pgc9s8qqaq=dirpe0xb9q8qiLsFr0=vr0=vr0dc8meaabaqaciaacaGaaeqabaqabeGadaaakeaacuWG1bqDgaqeamaaBaaaleaacqWGIbGyaeqaaOGaeyypa0ZaaSaaaeaadaaeWbqaaiabcIcaOiabdAha2naaBaaaleaacqWGPbqAcqGGSaalcqWGIbGyaeqaaaqaaiabdMgaPjabg2da9iabigdaXaqaaiabdkfasbqdcqGHris5aOGaeiOkaOIaemyDau3aaSbaaSqaaiabdMgaPjabcYcaSiabdkgaIbqabaGccqGGPaqkaeaadaaeWbqaaiabdAha2naaBaaaleaacqWGPbqAcqGGSaalcqWGIbGyaeqaaaqaaiabdMgaPjabg2da9iabigdaXaqaaiabdkfasbqdcqGHris5aaaakiabcYcaSaaa@513F@

where

• *u*_*i*, *b *_represents the observed HUI3 utility score for the *i*^th ^individual reporting chronic condition(s) *b*; and

• *v*_*i*,*b *_is defined as described in the first step above

Note that the average theoretical HUI3 scores were age-standardized to the total Canadian population in the same manner, assuming that the population of theoretical co-morbid cases had the same age-sex structure as that of their observed counterparts.

## References

[B1] Coebergh JWW, Janssen-Heijnen MLG, Post PN, Razenberg PPA (1999). Serious co-morbidity among unselected cancer patients newly diagnosed in the Southeastern part of the Netherlands in 1993–1996. J Clin Epidemiol.

[B2] Fillenbaum GG, Pieper CF, Cohen HJ, Cornoni-Huntley JC, Guralnik JM (2000). Co-morbidity of five chronic health conditions in elderlycommunity residents: determinants and impact on mortality. J Gerontol Med Sci.

[B3] Gijsen R, Hoeymans N, Schellevis FG, Ruwaard D, Satariano WA, van den Bos GA (2001). Causes and consequences of co-morbidity: A review. J Clin Epidemiol.

[B4] Broemeling A-M, Watson D, Black C (2005). Chronic conditions and co-morbidity among residents of British Columbia. Centre for Health Services and Policy Research.

[B5] US Bureau of the Census (1992). International population reports, an aging world II. P25, 92-3.

[B6] Schellevis FG, van den Bos GAM, Tijssen JGP, Grobbee DE, Heinsbroek RPW (1997). Co-morbidity and chronic diseases. Report of the workshop 'Co-morbidity and chronic diseases.'.

[B7] Cameron CM, Purdie DM, Kliewer EV, McClure RJ (2005). Differences in prevalence of pre-existing morbidity between injured and non-injuredpopulations. Bulletin of the World Health Organization.

[B8] Murray CJL, Salomon JA, Mathers CD, Lopez AD, Eds (2002). Summary measures of population health: Concepts, ethics, measurement and applications.

[B9] Murray CJL, Lopez AD, Eds (1996). The global burden of disease: A comprehensive assessment of mortality and disability from diseases, injuries, and risk factors in 1990 and projected to 2020.

[B10] Stouthard MEA, Essink-Bot ML, Bonsel GJ (1997). Disability weights for diseases in the Netherlands.

[B11] Murray CJL, Lopez AD (2000). Progress and directions in refining the global burden of disease approach: A response to Williams. Health Economics.

[B12] Mathers C, Vos T, Stevenson C (1999). The burden of disease and injury in Australia. A Australian Institute of Health and Welfare(cat no. PHE17).

[B13] Vos T, Begg S (2000). The Victorian Burden of Disease Study: Morbidity.

[B14] Mathers CD, Sadana R, Salomon JA, Murray CJL, Lopez AD (2001). Healthy life expectancy in 191 countries, 1999. Lancet.

[B15] Mathers CD, Murray CJL, Salomon JA, Murray CJL, Evans D (2003). Methods for measuring healthy life expectancy. Health systems performanceassessment: debates, methods and empiricism.

[B16] World Health Organization (2003). World Health Report 2000, Health Systems: Improving Performance.

[B17] Mathers CD, Iburg KM, Begg SB (2006). Adjusting for dependent comorbidity in the calculation of healthy life expectancy. Population Health Metrics.

[B18] Schneeweis S, Seeger JD, MacClure M, Wang PS, Avorn J, Glynn RJ (2001). Performance of co-morbidity scores to control for confounding in epidemiologic studies using claims data. Am J Epidemiol.

[B19] Mathers CD, Bernard C, Iburg KM, Inoue M, Ma Fat D, Shibuya K, Stein C, Tomijima N, Xu H (2003). Global Burden of Disease in 2002: data sources, methods and results.

[B20] Béland Y (2002). Canadian Community Health Survey: methodological overview. Health Reports.

[B21] Feeny DH (2005). The Health Utilities Index: a tool for assessing health benefits. QoL Newsletter.

[B22] Furlong WJ, Feeny DH, Torrance GW, Barr RD (2001). The Health Utilities Index (HUI) system for assessing health-related quality of life in clinical studies. Ann Med.

[B23] Feeny D, Furlong W, Torrance GW, Goldsmith CH, Zhu Z, DePauw S, Denton M, Boyle M (2002). Multi-attribute and single-attribute utility functions for the Health Utilities Index Mark 3 system. Med Care.

[B24] Manuel DG, Luo W, Ugnat A-M, Mao Y (2003). Cause-deleted health-adjusted life expectancy of Canadians with selected chronic conditions. Chronic Diseases in Canada.

[B25] Rao JNK, Wu CFJ, Yue K (1992). Some recent work on resampling methods for complex surveys. Survey Methodology (Statistics Canada, Catalogue 12-001).

[B26] Rust KF, Rao JNK (1996). Variance estimation for complex surveys using replication techniques. Statistical Methods in Medical Research.

[B27] Murray CJL, Murray CJL, Lopez AD (1996). Rethinking DALYs. The global burden of disease: A comprehensive assessment of mortality and disability from diseases, injuries, and risk factors in 1990 and projected to 2020.

[B28] Andrews G, Sanderson K, Beard J (1998). Burden of disease: methods of calculating disability from mental disorder. British Journal of Psychiatry.

[B29] Beckett M, Weinstein M, Goldman N, Yu-Hsuan L (2000). Do health interview surveys yield reliable data on chronic illness among older respondents?. Am J Epidemiol.

[B30] Gross R, Bentur N, Elhayany A, Sherf M, Epstein L (1996). The validity of self-reports on chronic disease: characteristics of underreporters and implications for the planning of services. Public Health Rev.

